# Comparison of the effects of transcranial direct current stimulation and mindfulness-based stress reduction on mental fatigue, quality of life and aggression in mild traumatic brain injury patients: a randomized clinical trial

**DOI:** 10.1186/s12991-021-00355-1

**Published:** 2021-06-15

**Authors:** Sheida Shirvani, Mohammadreza Davoudi, Masoud Shirvani, Peiman Koleini, Safora Hojat Panah, Fatemeh Shoshtari, Abdollah Omidi

**Affiliations:** 1 Department of Clinical Psychology, School of Medicine, Kashan University of Medical Sciences, Kashan, Iran; 2 Department of Clinical Psychology, Faculty of Behavioral Science, University of Social Welfare and Rehabilitation Sciences, Tehran, Iran; 3 Department of Neurosurgery, Milad Hospital, Tehran, Iran; 4 Department of Dental Surgery, School of Dentistry, Isfahan (Khorasgan) Branch, Islamic Azad University, Isfahan, Iran; 5 Department of Clinical Psychology, University of Najaf Abad, Isfahan, Iran

**Keywords:** Traumatic brain injuries, Mindfulness-based stress reduction, Transcranial direct current stimulation

## Abstract

**Background:**

The rate of traumatic brain injuries (TBIs) due to the accidents is high around the world. Patients with mild TBIs may suffer from some psychological disorders, including aggression, and mental fatigue, and thus their quality of life decreased. Among different treatments for TBI, two treatments, namely transcranial direct current stimulation (tDCS), and mindfulness-based stress reduction (MBSR) have shown to be effective. Therefore, this study aimed to compare the effects of these two treatments on mental fatigue, aggression and quality of life in mTBI patients.

**Materials and methods:**

This randomized controlled trial study was conducted on 48 TBI patients referred to emergency and neurosurgery departments of Shahid Beheshti Hospital, Kashan, Iran. They were selected using the convenience sampling method. Data were collected using the mental fatigue scale, the World Health Organization Quality of Life-BREF (short version), and the Buss–Perry Aggression Questionnaires. Then, the data were analyzed using a Mixed Repeated Measures ANOVAs, and the Levene and Kolmogorov–Smirnov tests by SPSS-23 software.

**Results:**

The mean age of patients in the three groups of MBSR, tDCS and control were 69.38 + 6.11 (25% male), 25.40 + 12.11 (25% male) and 69.37 + 0.2 (18.8% male), respectively. There was no significant difference between the three groups in terms of mental fatigue, quality of life and aggression (*P* < 0.05). In addition, the results showed that there was a significant difference between the main effect of time and the interaction between time and group (*P* < 0.001).

**Conclusions:**

Both MBSR and tDCS methods are effective in reducing the mental fatigue and aggression and increasing quality of life of mTBI patients; MBSR treatment, as indicated in the present study, can be more effective than tDCS in patients with mTBI.

*Trial registration* : Thailand Registry of Clinical Trials, TCTR20180827003 Registered on August 24, 2018.

## Background

Traumatic brain injuries (TBIs), which have been referred to the silent epidemic contribute to worldwide mortality and disability more than any other trauma-related injuries. 69 million persons incur TBI from all causes every year [1]. Iran with 429 TBI patients per 10,000 individuals is one of the countries with the highest rate of traumatic accidents [2]. About 75% of these injuries involve concussions or some other form of injury, which are regarded as mild traumatic brain injuries (mTBI) [3]. Among various clinical presentations of chronic mTBI, aggression and mental fatigue are two consequences that these patients experienced.

Aggression is one of the most common consequences of mTBI and its prevalence rates range from 11 to 34%. Aggression can impede rehabilitation and is a major cause of burden both to the patient and caregivers [4]. One of the most prevalent complaints after mTBIs, during the acute period, is mental fatigue, which is the most severe symptom. The incidence rates of mental fatigue after TBI range from 21 to 73% [5]. These adverse psychosocial consequences continued 10–20 years after TBI and such deficits will possibly continue throughout lifetime and can affect quality of life [6]. Beside many researches about mTBI, there is no effective treatment targeting aggression, mental fatigue and quality of life in these patients. Snell and colleagues in 2009 [7] systematically reviewed the treatments related to mTBI and found there is no robust effective treatment for mTBI. Findings of some studies have shown that two different treatments, namely Mindfulness-Based Stress Reduction (MBSR) and transcranial Direct Current Stimulation (tDCS) can improve some mental problems related to TBI.

Mindfulness-based stress reduction, which is a group-based intervention designed for patients with chronic pain, has been extensively used in medical and psychiatric populations including those with chronic fatigue, pain, psoriasis, and even cancers [1]. Mindfulness involves mastering the power of focus and fostering moment-to-moment awareness of thoughts, feelings and perceptions about the body. When patients are more aware of their emotions and body sensation, their overall capacity to track and cope with stress is increasing. Some studies demonstrated effectiveness of MBSR on mental fatigue, quality of life and aggression among various medical and psychological conditions [8, 9]. However, as these problems somewhat have been neglected in TBI patients, there is only one RCT study about examination of MBSR on these symptoms among TBI patients [10] and the results are limited.

Transcranial direct current stimulation is a safe and non-invasive form of neuro-modulation in which a low, direct current is applied to the skull through anodal and cathodal, reaching the cortical areas and modulating the resting membrane potential of individual neurons [11]. tDCS is often used to improve cognitive functioning in individuals with brain injury and patients with Parkinson’s disease [12]. In addition, regarding aggression, anodal tDCS over 15 days can ameliorate mental fatigue symptoms in patients who have had a post-polio syndrome and other populations, but there is no strong evidence on efficacy of tDCS in TBI patients [13].

Overall, due to the importance of treating these symptoms in the recovery process, preferential treatment should be specified in a controlled trial setting. Therefore, the aim of this study was to compare tDCS and MBSR in mental fatigue, quality of life and aggression in mTBI patients.

## Methods

### Participants

This randomized controlled trial study was conducted on 48 TBI patients hospitalized in emergency and neurosurgery departments of Shahid Beheshti Hospital, Kashan, Iran, in 2017. They were selected using the convenience sampling method [14].

### Sample size

With regard to limitations in sampling and rehabilitation of patients with brain damage, considering the 95% confidence and 80% test capability, using the following formula, the number of samples in each group were calculated (*n* = 16). For sample size calculation, we used standard deviations from a similar paper that has been conducted in food craving:

$$n = \frac{{\left( {z_{{1 - \frac{\alpha }{2}}} + z_{{1 - \beta }} } \right)\left( {\sigma _{{1{\text{~}}}}^{2} {\text{ + }}\sigma _{{2{\text{~}}}}^{2} } \right)}}{{\left( {M_{1} - M_{2} } \right)^{2} }}$$

### Selection criteria

All the patients were given a written explanation of the study protocol and were invited to participate in the project if they met the following criteria: (1) 18–50 years old; (2) a Glasgow Coma Scale score between 13 and 15; (3) a post-traumatic amnesia (PTA) more than 1 h; (4) localized or disseminated brain damage made by an external mechanical force; (5) brain imaging findings such as skull fracture or acute brain injury; (6) no history of substance abuse or previous neurological psychiatric disorders; and (7) informed consent to take part in the project.

Exclusion criteria included (a) lack of willingness to continue the research; (b) the absence of more than one session; (c) starting a secondary therapy; and (d) the use of other substances (except alcohol, and caffeine) during all stages of research.

### Randomization and procedure

From a total of 322 patients with TBI, 48 cases were eligible to participate in the study, based on the inclusion criteria. A list of these 48 people was prepared. Then, they were assigned to three groups (tDCS, MBSR, and control groups) using simple random allocation. This randomization was performed separately for each group.

To handle the intervention sessions, each of the tDCS and MBSR groups was divided into two groups. Both MBSR and tDCS therapies consisted of eight sessions. Therefore, both intervention groups received eight-session interventions in two groups of eight (a total of four groups of eight for both interventions). The control group did not receive any intervention and was studied only in the evaluation phases.

Patients were reassessed at baseline, post-treatment (immediately after the last treatment session), and 2 months after the treatment. Before starting the intervention, both groups received explanations about the content of the intervention. Participants were asked to raise any concerns or queries regarding the intervention, and interveners answered their queries clearly and provided information for them. Participants in each group were unaware of the existence of the other groups. They were blinded to the study aims and hypotheses. In addition, they only received necessary information about their treatment.

### Measurements

#### Mental fatigue scale

The mental fatigue scale (MFS) is a multidimensional self-report questionnaire with 15 items. It consists of cognitive, psychological, sensitive symptoms, and sleep quality subscales. This questionnaire was prepared by Johnson et al. in 2009–2010 to examine the dimensions of mental fatigue in patients with neurological disorders, and its Cronbach’s alpha coefficient was reported to be 0.9. The score range is from 0 to 42, and higher scores reflect a more severe symptom [15, 16]. The validity and reliability of the Persian version of the scale were assessed. In the current study, the MFS showed a high internal consistency (*α* = 0.91) [17].

#### Quality of life

The World Health Organization Quality of Life-BREF (short version) (WHOQOL-BREF) was developed for measuring quality of life. In Iran, it has been translated and standardized according to scientific principles, and its reliability and validity have been approved to be used in Persian population. Internal consistency was measured using Cronbach’s alpha of the four aspects, which was 0.77 in the patient group and 0.73 in the healthy group. The questionnaire consists of 26 items. Participants’ scores range between 4 and 20 [18].

#### Aggression scale

The new version of the Buss–Perry Aggression Questionnaire (BPAQ) is a self-rating scale which has 29 items answered on a five-point Likert and has four subscales of physical aggression (PA), verbal aggression (VA), anger (A) and hostility (H). The results of the test–retest coefficients for the four subscales (9 weeks apart) were 80.0–72.0 and the correlation between the four subscales ranged 38.0–49.0. The internal validity of the scale was measured using Cronbach’s alpha coefficient; these coefficients for the internal consistency of the subscales of physical aggression, verbal aggression, anger, and hostility were 82.0, 81.0, 83.0, and 80.0, respectively [18].

### Treatments

#### tDCS

The tDCS electrical stimulation was delivered using a tap-water soaked sponge pair of rubber carbon pads (each 10.5 cm^2^). The pads were fixed by rubber bands on the heads of the participants. Anodal stimulation (1.5 mA, 20 min) was carried out on (left frontal areas) F3 through ten sessions of tDCS treatment (three times a week). According to the existing studies and the opinions of some experts, after considering the two main points of the protocol, the amount of electric current from 1 to 2 mA during ten sessions (three sessions of 20 min per week) were considered. During the sessions, the amount of current was determined and electricity was increased [19, 20].

The cathodal stimulation was fixed over (right DLPFC) FP2. Both anodal and cathodal stimulations were delivered by an electrical stimulator tDCS device (Active dose II). Safety guidelines specified by Nitsche et al. [21] were taken into consideration [22]. The participants were informed that this treatment would not be considered as a first-line treatment for TBI. The related information sheets included the explanation for frequent adverse effects of tDCS (itching and tingling skin sensation, skin reddening, and headache). With regard to group separation (sham and active), no differences in information were presented. The sham tDCS mode started with a variable ramp-in and ramp-out phases. This was followed by an impedance control mode with small measuring pulses of 100e200 mA amplitude every 400e550 ms for the same period as in the active condition, and ended with another ramp-in and ramp-out phase. The tDCS was carried out passively and the participants did not engage in an online task.

#### MBSR

MBSR was delivered by two doctoral clinical psychologists who had completed their 1-year full-time MBSR training in Kashan University of Medical Sciences. They were blinded to the existence of other groups as well as the study aims. They implemented an 8-week program of mindfulness meditations developed by Jon Kabat-Zinn (1996). Groups of eight patients met for eight sessions each session lasted for 2 h. To support the practice, each participant was presented with a MBSR workbook. The workbook included descriptions for mindfulness exercises. It also included prerecorded audio files to support ongoing practice [23]. The eight therapy sessions followed the program outlined in Table 1.

**Table 1:** MBSR sessions

Session	Content
Pre-session and preparation	Seeking familiarity with participants and make a rapport. Discussion about treatment and MBSR approach. Trying to identify obstacles and solving them
1	Body scan, mindful breathing, and mindful eating
2	Body scan, mindful breathing, mindful eating, and mindful tooth brushing
3	Body scan, sitting meditation, completing pleasant events calendar, continuing infuse mindfulness into daily life activity
4	Everyday yoga, STOP technique, continuing infuse mindfulness into daily life activity
5	Body scan, yoga, and sitting meditation in alteration, brain and meditation, loving kindness meditation, completing communication calendar during week, continuing infuse mindfulness into daily life activity
6	Body scan, yoga, and sitting meditation in alteration, introducing conflict resolution styles, AH-FOWL exercise, continuing infuse mindfulness into daily life activity
7	Body scan, yoga, and sitting meditation in alteration, pain process, learning about emotions, continuing infuse mindfulness into daily life activity
8	Body scan, yoga, and sitting meditation in alteration, continuing infuse mindfulness into daily life activity, writing about short-time and long-time goals

#### Data analysis

Data were analyzed using descriptive statistics (mean, frequency and standard deviation), mixed Repeated Measures ANOVAs, and Levene and Kolmogorov–Smirnov tests by the SPSS software version 23. At first, clinical variables were examined for normality to use parametric or nonparametric analyses. Levene and Kolmogorov–Smirnov were used to investigate the normality and homogeneity of the distribution. Then, as can be seen in the results, the variables did not meet the assumptions of nonparametric tasting. Therefore, parametric analyses were used. Moreover, descriptive tests including Chi-square and one-way ANOVA were used to compare the means and frequencies of variables. In addition, mixed Repeated Measures ANOVAs were used to evaluate the effectiveness of all three groups during the review phases and compare this effectiveness for clinical variables. The least significant deviation (LSD) test was also applied to compare the three groups.

### Ethical considerations

All participants were asked to complete an informed consent from prior to participation in the study. This study was approved by the Ethics Committee of Kashan University of Medical Sciences (IR.KAUMS.NUHEPM.REC.1396.12). Moreover, the registered code in the RCT system is TCTR20180827003.

## Results

**Fig. 1:**
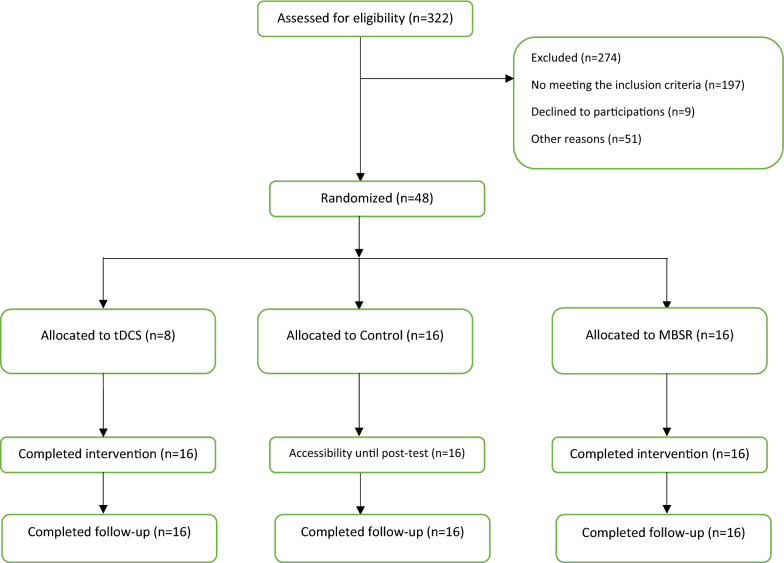
Flow chart diagram

In the present study, three groups of patients participated in the control group and two intervention groups of tDCS and MBSR. The dropout rate was 0% and all subjects continued treatment until the end. Figure 1 demonstrates consort diagrams.

The results revealed that there was no significant difference in education and age of male and female patients with brain injury (*P* > 0.05). The participant’s demographic information is presented in Table 2.

**Table 2:** Demographic characteristics of participants

Variables		tDCS number (%)	MBSR number (%)	Control number (%)	*X*^2^	*P* value
Gender (*N*)	Female	12 (75)	12 (75)	13 (81.2)	0.239	0.889
	Male	4 (25)	4 (25)	3 (18.8)		
Education (*N*)	Middle school degree	2 (12.5)	1 (6.2)	3 (18.8)	0.615	8.14
	Diploma	9 (56.2)	7 (43.7)	5 (31.2)		
	Associate degree	0 (0)	0 (0)	1 (6.2)		
	Bachelor	3 (18.8)	5 (31.2)	6 (37.5)		
	Master	2 (12.5)	2 (12.5)	0 (0)		
	PhD	0 (0)	1 (6.2)	1 (6.2)		
Total		16 (100)	16 (100)	16 (100)		
Age (M ± SD)		40.25 ± 11.12	38.69 ± 11.63	37.69 ± 10.29	F	*P* value
					0.219	0.804
BMI		25 ± 2.42	24.4 ± 2.6	23.3 ± 2.3	1.8	0.166

The results showed that all research variables were in normal distribution using the Kolmogorov–Smirnov test. Furthermore, in post-test and follow-up, Levene’s test was not significant for none of the variables. The results of ANOVA showed no significant baseline differences between the three groups (*P* > 0.05). As reported in Table 3, no differences were found between the groups regarding mental fatigue, quality of life, and aggression (*P* > 0.05).

**Table 3:** Mean and standard deviations of research variables

Time	Dependent variable	tDCS	MBSR	Control	*F*	*P*
Pre-test	Mental fatigue	24.22 ± 3.56	25.28 ± 4.12	24.28 ± 5.20	1.5	0.223
	Quality of life	81.19 ± 10.47	69.00 ± 12.81	73.00 ± 11.04	0.19	0.826
	Aggression	91.12 ± 20.92	107.00 ± 21.91	94.87 ± 23.98	1.1	0.340
Post-test	Mental fatigue	14.44 ± 3.84	9.44 ± 4.29	26.84 ± 3.77	81.4	*P* < 0.001
	Quality of life	86.06 ± 12.93	97.69 ± 9.43	71.31 ± 8.11	26.03	*P* < 0.001
	Aggression	79.87 ± 21.04	65.75 ± 13.80	105.56 ± 14.48	23.19	*P* < 0.001
Follow-up	Mental fatigue	14.94 ± 4.28	6.91 ± 5.38	26.47 ± 3.72	75.9	*P* < 0.001
	Quality of life	85.37 ± 11.67	100.31 ± 9.21	72.19 ± 7.70	33.8	*P* < 0.001
	Aggression	79.06 ± 21.40	65.50 ± 14.80	106.69 ± 12.58	28.6	*P* < 0.001

### Mental fatigue

**Fig. 2:**
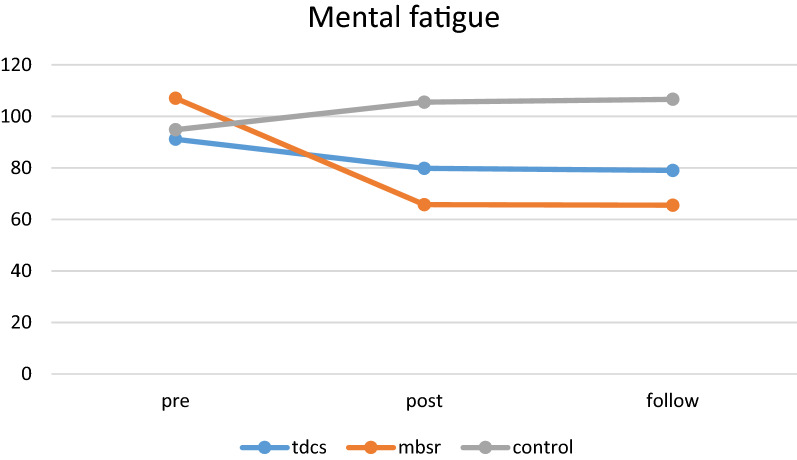
Mental fatigue trends during assessments

Repeated measure analysis showed that both time (*P* < 0.001) main effect and interaction between time × group (*P* < 0.001) were significant (Table 4). As reported in Table 4, there was a significant difference in MFS from pre-test to follow-up periods. Generally, 84% of variation can be explained by the MFS score (Time effect). In pairwise comparisons (LSD test), our findings showed that MFS were significantly decreased from pre- to post-phase and from pre- to follow-up phase in both tDCS and MBSR groups (*P* < 0.001) and we found significant changes in the follow-up stage in the MBSR group (*P* = 0.005) (Fig. 2). In addition, the rate of changes in mental fatigue was significantly reduced in the MBSR group compared to the tDCS and control groups (*P* < 0.01). Mental fatigue was significantly reduced in the tDCS group compared to the control group (*P* < 0.001).

**Table 4:** Repeated measure results

Dependent variable	Source	SS	df	MS	*F*	*P* value	Eta
Mental fatigue	Time	2109.02	1.79	1175.55	249.44***	0.001	0.847
	Time * group	2096.99	3.59	584.42	124.01***	0.001	0.846
Quality of life	Time	2959.37	2.20	3304.01	69.95***	0.001	0.609
	Time * group	5941.33	2.40	2478.95	52.48***	0.001	0.700
Aggression	Time	6683.51	1.20	5559.96	32.54***	0.001	0.420
	Time * group	15823.82	2.40	6581.86	38.52***	0.001	0.631

*Df* degree of freedom, *Ss* sum of squares, *Ms* mean of squares, *F* f-statistic, *Eta* eta-squared

**significant at the 0.05 level (2-tailed)

### Quality of life

**Fig. 3:**
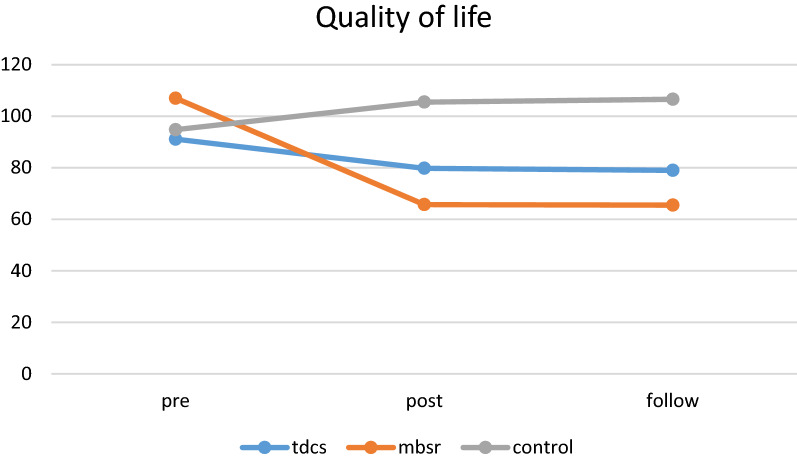
Quality of life trends during assessments

Repeated measure analysis showed that both time as a main effect (*P* < 0.001) and interaction between time × group (*P* < 0.001) were significant (Table 4). As reported in Table 4, there was a significant difference in the quality of life from pre-test to follow-up periods. Generally, 60% of the variation can be explained by the quality of life score (time effect). In time pairwise comparisons (LSD test), our findings showed that the mean change in quality of life in the MBSR group had a significant increase from pre-test to post-test stage and from pre-test to follow-up as well as from post-test to follow-up stage (*P* < 0.01). In the tDCS and control groups, there was no significant difference in the mean quality of life from the pre-test to post-test stage (*P* < 0.05) (Fig. 3). In addition, the mean difference was not significant in the MBSR group compared to the tDCS group (*P* = 0.165).

### Aggression

**Fig. 4:**
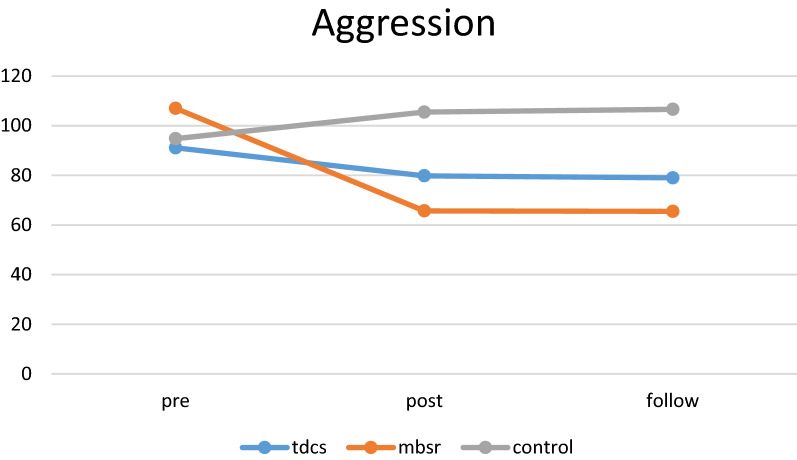
Aggression trends during assessments

Repeated measure analysis showed that both time main effect (*P* < 0.001) and interaction between time × group (*P* < 0.001) were significant (Table 4). As reported in Table 4, there is significant difference in aggression from pre-test to follow-up periods. Generally, 42% of variation can be explained by aggression score (time effect). In pairwise comparisons (LSD test), the findings of the present study showed that aggression was decreased from pre- to post-test phase and from pre-test to follow-up phase in both tDCS and MBSR groups (*P* < 0.001). In addition, there was a significant change in follow-up stage in the MBSR group (*P* = 0.014) (Fig. 4). In addition, the mean differences were not significant comparing both MBSR and tDCS groups (*P* = 0.412).

## Discussion

The current study aimed at comparing the effects of the tDCS and MBSR on mental fatigue, quality of life and aggression in patients with mTBI. This study was the first RCT study conducted to examine three rehabilitation methods on TBI patients. Regarding mental fatigue, the results showed that mental fatigue was decreased more significantly in both intervention groups compared to the control group. In addition, mental fatigue in the MBSR group decreased more significantly compared to the tDCS group. Additionally, no significant difference was found in the scores of quality of life and aggression between the two intervention groups. However, aggression was reduced and quality of life was increased more significantly in the two intervention groups compared to the control group.

This study demonstrated a significant effect of the third wave of psychotherapy and the use of neuroscience equipment to rehabilitate TBI patients and it has been shown that although routine rehabilitation, which is often a form of pharmacological intervention, can be helpful, it does not meet all the needs of TBI patients, which is mainly due to the complications of brain damage, and if left untreated, can lead to widespread limitations in various physical, cognitive, social, and psychological dimensions.

Results showed clinically significant improvements in perceived self-efficacy among the participants, especially for the improvement of cognitive and emotional symptoms. In addition, findings of some other studies evinced that after the intervention participants displayed a more positive orientation to problem-solving. Such improvements in perceived self-efficacy and orientation to problem-solving may lead to the global life satisfaction that can also be associated with the intervention. These findings are consistent with the finding of Bédard et al. [24]. Regarding the effect of mindfulness training on psychological variables in nonneurologic samples, we observed the big effect size in other studies which is in line with the current meta-analysis [25, 26]. Contrary to existing explanations for the direct impact of MBSR treatment on improving the quality of life, previous research has acknowledged that tDCS can improve the quality of life of the patients due to its therapeutic effects on other psychological variables [12, 26]. Given that the variable of mental fatigue includes the basic dimensions used in patients’ daily lives and the effect size of tDCS on MFS was high, it can be stated that tDCS can have a significant therapeutic effect on MFS and can improve the quality of life of TBI patients [26].

Another notable finding from this study is that MBSR and tDCS are useful with TBI. There are many reasons for this. MBSR showed a significant effect on mitigating aggression. Mindfulness-based therapies can be helpful for clients to focus on their attention, be non-judgmental and accepting, and be present in the moment. In conceptual review, explanations for the evident success of mindfulness-based therapies are emphasized; there are several mechanisms that can indicate how mindfulness strategies change behavior [27]. These skills include exposure (to unpleasant experiences), cognitive change, and self-control. Mindfulness therapies may operate like CBT, which is a substantiated treatment for controlling anger, using cognitive skills that finally result in cognitive change. Mindfulness-based treatments are also unique in that they are not reliant on the participation of a secondary member in treatment, and may be preferred by independently oriented clients struggling with aggression problems [27].

In the present study, a new approach (tDCS) was tested to decrease aggressive behaviors. The probability of performing aggressive behaviors was decreased in people who underwent bilateral anodal stimulation of the DLPFC using tDCS. The treatment aggressive intent relationship was partly accounted for by enhanced perception that the aggressive act were more morally wrong, resulting from prefrontal up regulation, findings help to strengthen conclusions from neurological, neuroimaging, and neuropsychological research [28]. By documenting experimentally, the role of the prefrontal cortex on the likelihood of engaging in aggression and the perception of such acts as morally wrong.

Beyond examining the role of the prefrontal cortex on a behavioral symptom, the finding showed that moral judgment partly mediates the effect of tDCS. It also provides partial support for the neuromoral theory of aggressive behavior, which postulates that moral cognition and emotion [29].

Another interesting finding from this study is that MBSR and tDCS were useful and had a notable and big effect size. This result is in line with the study by [10]. According to mental fatigue theories, cognitive activities needed more resources than normal and lead to a greater neural activity in comparison to controls during a given mental activity [30, 31]. This indicates an increased cerebral effort after brain injury. One reason why MBSR was effective may be that this treatment offers strategies to better handle stressful situations and economize with mental energy. Meditation techniques in healthy subject were suggested to improve attention performances, processing speed and cognitive flexibility [32]. MBSR is also associated with changes in brain activity involved in attention [22]. Subjects with mental fatigue have difficulties within these domains and will easily become even more fatigued if the activity is not adapted to their capabilities. It is, therefore, interesting to see that MBSR seems to increase attention and also processing speed. Mental fatigue may be caused by a dysfunction or imbalance in the signaling system(s) in the brain and that the brain works with less precision [30]. Improvements in the neural network may have been achieved during the course of this study. The findings of the current study show that the tDCS had a significant effect on the improvement of mental fatigue and the component of mental fatigue is among the cognitive functions of the brain [5]; this result is consistent with the findings of other similar studies [12].

Non-invasive neuromodulatory tDCS can modulate cortical excitability and enhance the effects of cognitive training, and thus tDCS could be used to supplement cognitive training [16]. Regarding the mechanism of tDCS, it has been suggested that tDCS depolarizes or hyperpolarizes the membrane potential of the brain tissue and hence induces changes in brain excitability. Rango et al. reported the interesting finding that anodal tDCS over the frontal lobe induced a significant increase in myo-inositol content below the stimulating electrode in a proton magnetic resonance spectroscopy study. Therefore, it is probable that current changes in the tissue induced by tDCS secondarily causes neurochemical change in the brain [12].

### Limitations and future direction

Primarily, issues such as a larger sample size, double-blind designs and control of previous psychopathological symptoms would be desirable in the current study. Second, adverse effect has not been reported. In addition, taking quantitative electroencephalography (qEEG) before applying electrical stimulation could have helped us choose the best stimulation site for each patient. The qEEG-based location of stimulation can be more specific and sensitive. In addition, parallel control groups were not used for every intervention arm, which may lead to a systematic bias. Since this study was the first one comparing MBSR and tDCS, further studies with larger sample sizes and controlling interfering variables are needed to duplicate our results. Finally, the future studies can assess adverse effects for every treatment and also assess combination of MBSR and tDCS as a one arm to assess complementary efficacy.

### Clinical implication

This study provides promising evidence that the use of MBSR can be helpful in mTBI patients. These findings presented the widespread acceptance and interest in alternative and complementary treatments like meditation among patients recovering from TBI. In fact, patients with mTBI, as a post-treatment feedback, were very positive and consistently reported some benefits of the interventions that affected and improved their life; even those who have doubt about the alternative treatments describe the treatments as being “life changing”. Many of their comments throughout the study were used to shape the final treatment product.

## Conclusions

The findings of the present study reveal that both MBSR and tDCS were effective in improving mental fatigue, quality of life and aggression compared to the control group. In addition, MBSR treatment has a more positive effect on psychological variables such as aggression and quality of life, while tDCS treatment has a better effect on cognitive variables like mental fatigue.

## Data Availability

Not applicable because this is a protocol.

## Abbreviations

mTBI: Mild traumatic brain injury
MBSR: Mindfulness-based stress reduction
tDCS: Transcranial direct current stimulation
MFS: Mental fatigue scale
WHOQOL-BREF: World Health Organization quality of life-BREF
BPAQ: Buss–Perry aggression questionnaire
qEEG: Quantitative electroencephalography

## References

[1] Dewan MC, Rattani A, Gupta S, Baticulon RE, Hung YC, Punchak M (2018). Estimating the global incidence of traumatic brain injury. J Neurosurg.

[2] Farzandipour M, Ghatan H, Mazrouei L, Nejati M, Agha BT. Epidemiological study of traumatic patients referred to neghavi hospital of kashan. 2007.

[3] Faul M, Xu L, Wald MM, Coronado VG. Traumatic brain injury in the United States. US department of health and human services, Centers for disease control and 2010. p. 2010.

[4] Rao V, Rosenberg P, Bertrand M, Salehinia S, Spiro J, Vaishnavi S (2009). Aggression after traumatic brain injury: prevalence and correlates. J Neuropsychiatr Clin Neurosci.

[5] Wylie GR, Flashman LA (2017). Understanding the interplay between mild traumatic brain injury and cognitive fatigue: models and treatments. Concussion.

[6] Hoofien D, Gilboa A, Vakil E, Donovick PJ (2001). Traumatic brain injury (TBI) 10? 20 years later: a comprehensive outcome study of psychiatric symptomatology, cognitive abilities and psychosocial functioning. Brain Inj.

[7] Snell DL, Surgenor LJ, Hay-Smith EJC, Siegert RJ (2009). A systematic review of psychological treatments for mild traumatic brain injury: an update on the evidence. J Clin Exp Neuropsychol.

[8] Lao SA, Kissane D, Meadows G (2016). Cognitive effects of MBSR/MBCT: a systematic review of neuropsychological outcomes. Conscious Cogn.

[9] Zhang Q, Zhao H, Zheng Y (2019). Effectiveness of mindfulness-based stress reduction (MBSR) on symptom variables and health-related quality of life in breast cancer patients–a systematic review and meta-analysis. Supportive Care Cancer.

[10] Johansson B, Rönnbäck L (2012). Mental fatigue and cognitive impairment after an almost neurological recovered stroke. ISRN Psychiatr.

[11] Datta A, Bikson M, Fregni F (2010). Transcranial direct current stimulation in patients with skull defects and skull plates: high-resolution computational FEM study of factors altering cortical current flow. Neuroimage.

[12] Kang E-K, Kim D-Y, Paik N-J (2012). Transcranial direct current stimulation of the left prefrontal cortex improves attention in patients with traumatic brain injury: a pilot study. J Rehabil Med.

[13] Acler M, Bocci T, Valenti D, Turri M, Priori A, Bertolasi L (2013). Transcranial direct current stimulation (tDCS) for sleep disturbances and fatigue in patients with post-polio syndrome. Restor Neurol Neurosci.

[14] Kekic M, McClelland J, Campbell I, Nestler S, Rubia K, David AS (2014). The effects of prefrontal cortex transcranial direct current stimulation (tDCS) on food craving and temporal discounting in women with frequent food cravings. Appetite.

[15] Johansson B, Ronnback L (2014). Evaluation of the mental fatigue scale and its relation to cognitive and emotional functioning after traumatic brain injury or stroke. Int J Phys Med Rehabil.

[16] Johansson B, Starmark A, Berglund P, Rödholm M, Rönnbäck L (2010). A self-assessment questionnaire for mental fatigue and related symptoms after neurological disorders and injuries. Brain Inj.

[17] Sheida Shirvani ZK, Koleini P, Fakharian E, Mosavi G, Omidi A (2020). Predicting the quality of life of patients with mild traumatic brain injury: a study based on psychological variables. Arch Trauma Res.

[18] Mehdizadeh Kashi A, Moradi Y, Chaichian S, Najmi Z, Mansori K, Salehin F (2018). Application of the World Health Organization quality of life instrument, short form (WHOQOL-BREF) to patients with endometriosis. Obstet gynecol Sci.

[19] Nitsche MA, Paulus W (2001). Sustained excitability elevations induced by transcranial DC motor cortex stimulation in humans. Neurology.

[20] Wang B, Xiao S, Yu C, Zhou J, Fu W (2021). Effects of transcranial direct current stimulation combined with physical training on the excitability of the motor cortex, physical performance, and motor learning: a systematic review. Front Neurosci.

[21] Nitsche MA, Fricke K, Henschke U, Schlitterlau A, Liebetanz D, Lang N, Henning S, Tergau F, Paulus W (2003). Pharmacological modulation of cortical excitability shifts induced by transcranial direct current stimulation in humans. J Physiol..

[22] Belmont A, Agar N, Hugeron C, Gallais B, Azouvi P (2006). Fatigue and traumatic brain injury. Ann Readapt Med Phys.

[23] Didonna F (2009). Clinical handbook of mindfulness.

[24] Bédard M, Felteau M, Mazmanian D, Fedyk K, Klein R, Richardson J (2003). Pilot evaluation of a mindfulness-based intervention to improve quality of life among individuals who sustained traumatic brain injuries. Disabil Rehabil.

[25] Grossman P, Niemann L, Schmidt S, Walach H (2004). Mindfulness-based stress reduction and health benefits: a meta-analysis. J Psychosom res.

[26] Kay T, Newman B, Cavallo M, Ezrachi O, Resnick M (1992). Toward a neuropsychological model of functional disability after mild traumatic brain injury. Neuropsychology.

[27] Del Vecchio T, O'Leary KD (2004). Effectiveness of anger treatments for specific anger problems: a meta-analytic review. Clin Psychol Rev.

[28] Raine A, Yang Y (2006). Neural foundations to moral reasoning and antisocial behavior. Soc Cognit Affect neurosci.

[29] Azouvi P, Couillet J, Leclercq M, Martin Y, Asloun S, Rousseaux M (2004). Divided attention and mental effort after severe traumatic brain injury. Neuropsychologia.

[30] Moore A, Malinowski P (2009). Meditation, mindfulness and cognitive flexibility. Conscious Cogn.

[31] Kohl AD, Wylie GR, Genova HM, Hillary FG, Deluca J (2009). The neural correlates of cognitive fatigue in traumatic brain injury using functional MRI. Brain Inj.

[32] Kilpatrick LA, Suyenobu BY, Smith SR, Bueller JA, Goodman T, Creswell JD (2011). Impact of mindfulness-based stress reduction training on intrinsic brain connectivity. Neuroimage.

